# High Expression of Interferon Pathway Genes CXCL10 and STAT2 Is Associated with Activated T-Cell Signature and Better Outcome of Oral Cancer Patients

**DOI:** 10.3390/jpm12020140

**Published:** 2022-01-21

**Authors:** Yun-Cian Huang, Jau-Ling Huang, Lu-Chia Tseng, Ping-Hung Yu, Si-Yun Chen, Chang-Shen Lin

**Affiliations:** 1 Graduate Institute of Medicine, College of Medicine, Kaohsiung Medical University, Kaohsiung 807, Taiwan; yuncian1103@gmail.com (Y.-C.H.); qoxoruby@gmail.com (S.-Y.C.); 2 Department of Bioscience Technology, College of Health Science, Chang Jung Christian University, Tainan 711, Taiwan; jaulingh@mail.cjcu.edu.tw (J.-L.H.); luke20000820@gmail.com (L.-C.T.); 3 Department of Nursing, National Taichung University of Science and Technology, Taichung 404, Taiwan; oxfordocean@gmail.com; 4 Center for Cancer Research, Kaohsiung Medical University, Kaohsiung 807, Taiwan; 5 Department of Medical Research, Kaohsiung Medical University Hospital, Kaohsiung Medical University, Kaohsiung 807, Taiwan; 6 Department of Biological Sciences, National Sun Yat-sen University, Kaohsiung 804, Taiwan

**Keywords:** biomarker, CXCL10, interferon, oral cancer, prognosis, STAT2, T cells

## Abstract

To improve the survival rate of cancer patients, biomarkers for both early diagnosis and patient stratification for appropriate therapeutics play crucial roles in precision oncology. Investigation of altered gene expression and the relevant molecular pathways in cancer cells are helpful for discovering such biomarkers. In this study, we explore the potential prognostic biomarkers for oral cancer patients through systematically analyzing five oral cancer transcriptomic data sets (TCGA, GSE23558, GSE30784, GSE37991, and GSE138206). Gene Set Enrichment Analysis (GSEA) was individually applied to each data set and the upregulated Hallmark molecular pathways of each data set were intersected to generate 13 common pathways including interferon-α/γ pathways. Among the 5 oral cancer data sets, 43 interferon pathway genes were commonly upregulated and 17 genes exhibited prognostic values in TCGA cohort. After validating in another oral cancer cohort (GSE65858), high expressions of C-X-C motif chemokine ligand 10 (CXCL10) and Signal transducer and activator of transcription 2 (STAT2) were confirmed to be good prognostic biomarkers. GSEA of oral cancers stratified by CXCL10/STAT2 expression showed that activation of T-cell pathways and increased tumor infiltration scores of Type 1 T helper (Th1) and CD8+ T cells were associated with high CXCL10/STAT2 expression. These results suggest that high CXCL10/STAT2 expression can predict a favorable outcome in oral cancer patients.

## 1. Introduction

Oral cancer includes the malignant cells derived from lips, tongue, inner cheeks, palate, and gums. The most common histological type of oral cancer is squamous cell carcinoma. According to GLOBOCAN statistics, there were 377,713 new lip and oral cancer cases and 177,757 new deaths worldwide in 2020 [1]. In the United States, the all-stages, 5-year survival rates range between 48% and 66% by all races. If diagnosed at advanced stage, the survival rates can be decreased by 20% from the average of all stages [2]. Thus, early diagnosis and proper anticancer treatment are required for improvement of patient survival. Investigation of cancer cell characteristics can help to identify good biomarkers for early diagnosis and to develop specific therapeutics for personalized treatment based on the feature of cancer cells. For examples, epidermal growth factor receptor (EGFR) is commonly overexpressed in head and neck cancer and thus the anti-EGFR therapeutics are used for the patients with head and neck cancer [3]. By examining gene expression signatures, a T cell-inflamed gene expression profile is found to be correlated with response to immunotherapy in multiple solid cancers including head and neck cancer [4]. For early diagnosis of oral cancer, many studies have demonstrated the potential use of cytokines, DNA methylation, and non-coding RNA including microRNAs in saliva as candidate biomarkers [5,6]; however, standardization in clinical practice is still lacking.

Interferons are members of innate cytokine and are involved in modulation of adaptive immune response [7]. Both innate and adaptive immunities play important roles in battling infections and malignancies [8,9]. Interferons regulate differentiation, maturation, and functions of various immune cells, such as natural killer (NK) cells, dendritic cells, B cells, helper (Th) and cytotoxic CD8+ T cells [7]. Moreover, interferons play an important role in modulating efficacy of anticancer immunotherapy [8,10]. Interferons are classified as types I (interferon-α/β), II (interferon-γ), and III (interferon-λ), each binds to its own receptor. Like viral nucleic acids in the cytosol of infected cells, genomic alterations in cancer cells may generate cytosolic DNA and induce abnormal RNA that can trigger the production of type I interferons. The secreted type I interferons bind to the heterodimeric receptors (IFNAR1/IFNAR2) and induce the expression of interferon-stimulated genes (ISGs) through Janus kinase 1 (JAK1)/Tyrosine kinase 2 (TYK2)-Interferon stimulated gene factor 3 (ISGF3) axis. ISGF3 is a trimeric transcriptional complex containing Interferon regulatory factor 9 (IRF9), Signal transducer and activator of transcription 1 (STAT1), and STAT2, which are phosphorylated by JAK1/TYK2 in response to interferon stimulation [7].

It is estimated that more than 2000 ISGs are induced in human primary fibroblasts by type I interferon stimulation. These ISGs including CXCL10, Interferon induced protein 44 like (IFI44L), Interferon induced with helicase C domain 1 (IFIH1), ISG15 ubiquitin like modifier (ISG15), STAT2, and so on [11]. Many studies have shown anticancer functions of interferons and ISGs. For examples, Makowska et al. report that interferon-β can induce NK cell-mediated apoptosis of nasopharyngeal carcinoma through upregulating TNF superfamily member 10 (TNFSF10/TRAIL) [12]. Overexpression of CXCL10 in HeLa cells leads to activation of TP53-mediated apoptosis [13]. Loss of STAT2 expression in Jurkat leukemia cells results in resistance to type I interferon-induced apoptosis [14]. However, the role of interferons and ISGs in oral cancer is not comprehensively evaluated. Here, we systematically examined the altered molecular pathways in oral cancer by analyzing 5 transcriptomic data sets and found significant activation of interferon-α and interferon-γ pathways. The prognostic values of the core enriched genes in interferon-α/γ pathways were examined in TCGA data set and were validated in another oral cancer cohort (GSE65858), which resulted in CXCL10 and STAT2. We demonstrated that high CXCL10/STAT2 expression was associated with activation of T-cell pathways, increased tumor infiltration of Th1 and CD8+ T cells, and better patient outcome.

## 2. Materials and Methods

### 2.1. Oral Cancer Data Sets

#### 2.1.1. Discovery Data Sets

The gene expression data in tumor and normal tissues of four oral cancer microarray data sets were retrieved from Gene Expression Omnibus with accession numbers GSE23558 (India), GSE30784 (USA), GSE37991 (Taiwan), and GSE138206 (China). Of note, 21 out of 27 samples (78%) in GSE23558 and all 40 cases (100%) in GSE37991 were from betel nut chewers [15,16]. The gene expression and clinical data of TCGA head and neck cancer (HNSC) were accessed in July 2020 by UCSC Xena cancer genome browser [17] and the 342 oral cancer data, which included cancers of alveolar ridge, base of tongue, buccal mucosa, floor of mouth, hard palate, lip, oral cavity, and tongue, were extracted from the whole HNSC data set. Table 1 shows the information of case numbers (tumor and normal control) and patient’s gender and age of the five oral cancer data sets. 

#### 2.1.2. Validation Data Sets

The gene expression and clinical data of 83 oral cancers were extracted from GSE65858 (Germany) data set to validate the prognostic roles of interferon pathway genes discovered in TCGA oral cancer data set. In addition to 83 oral cancers (cavum oris), GSE65858 data set also contains 197 laryngeal, hypopharyngeal, and oropharyngeal cancers. The patient characteristics including gender, age, T-stage, N-stage, survival, and smoking status of TCGA and GSE65858 oral cancer data sets are shown in Table 2.

### 2.2. Gene Set Enrichment Analysis

Gene set enrichment analysis (GSEA version 4.03, Broad Institute, Cambridge, MA, USA) [18] was performed for each oral cancer data set (tumor versus normal) to compute overlaps with the Hallmark gene sets in Molecular Signatures Database (MSigDB version 7.1, Broad Institute, Cambridge, MA, USA). The GSEA results were sorted by normalized enrichment score (NES) with nominal *p* value < 0.05 and false discovery rate < 0.25. For the patient subgroups stratified by CXCL10/STAT2 expression (HH versus LL), GSEA was run with the Hallmark and GO Biological Process ontology gene sets of MSigDB and the results of immune and T-cell pathways were presented. 

### 2.3. Kaplan-Meier Survival and Statistical Analyses

The comparison of patient characteristics between TCGA and GSE65858 cohorts was analyzed using chi square test. The patient overall survival (OS) in TCGA and GSE65858 oral cancer cohorts was analyzed by the web-based tools Kaplan-Meier plotter (http://kmplot.com/analysis/ accessed between 15 November 2020 and 4 April 2021) with “best cut-off” setting [19]. The IBM SPSS Statistics (version 22, Armonk, NY, USA) was used to compute hazard ratio (HR) of patient OS according to variables of CXCL10/STAT2 expression (HH versus LL), T-stage (T_3–4_ versus T_1–2_), N-stage (N_1–3_ versus N_0_), gender (male versus female), and age (>60-yr vs. <60-yr) by Cox model analysis. The adjusted HR was calculated by including the significant variables in univariate Cox regression analysis. 

### 2.4. Analysis of Tumor Infiltration Score

The tumor infiltration scores of various immune cells were examined using the web-based tools xCELL (https://xcell.ucsf.edu/ accessed on 4 April 2021), which performed cell type enrichment analysis based on specific gene expression signatures of different immune and stromal cells [20].

## 3. Results

### 3.1. Interferon Pathways Were Upregulated in Oral Cancer

**Figure 1:**
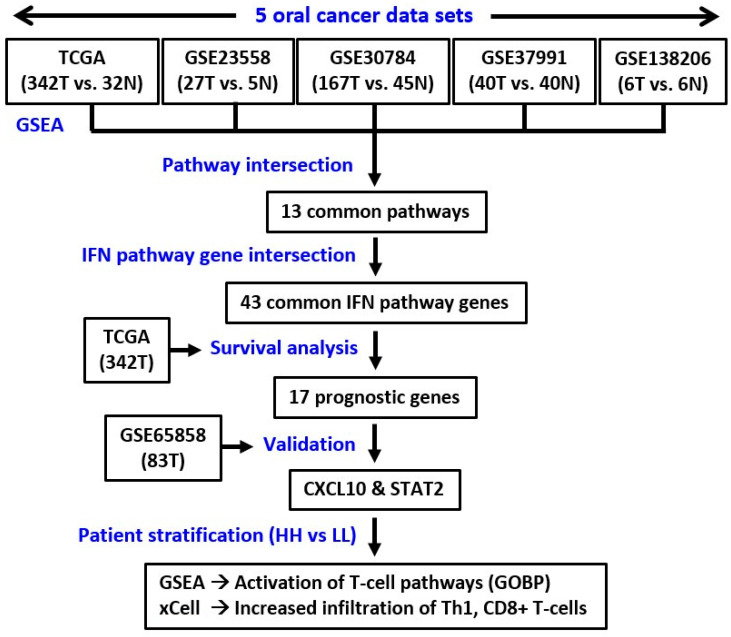
The study pipeline. T, tumor. N, normal. GSEA, gene set enrichment analysis. GOBP, Gene Ontology Biological Process.

**Figure 2:**
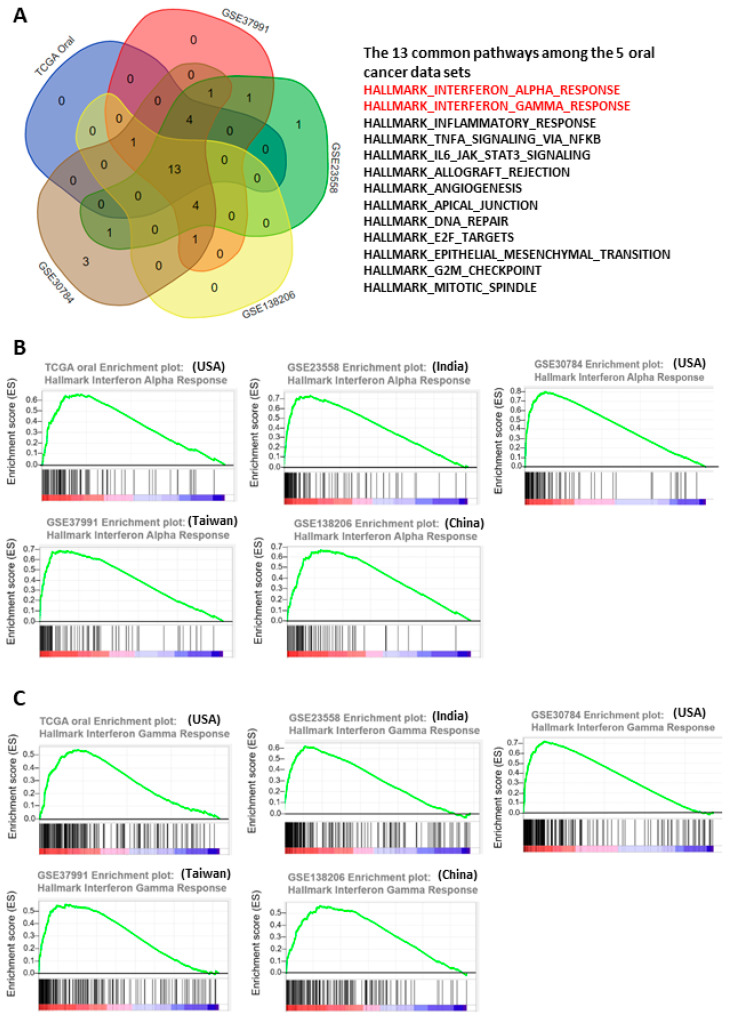
Activation of interferon pathways in the 5 oral cancer data sets. (**A**) Intersection of the upregulated molecular pathways among the 5 oral cancer data sets. (**B**,**C**) Enrichment plots of interferon-α (**B**) and interferon-γ (**C**) pathways in the 5 oral cancer data sets.

**Figure 3:**
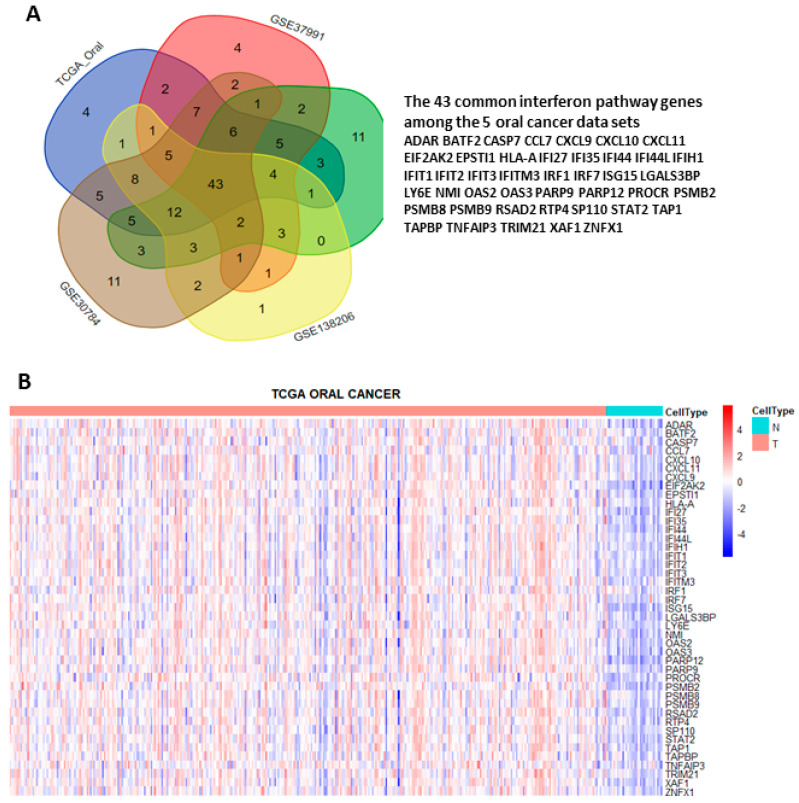
Identification of the 43 interferon pathway genes commonly upregulated in the 5 oral cancer data sets. (**A**) Intersection of the core enriched genes in interferon-α and interferon-γ pathways of each data set. (**B**) Heat map showing the differential expressions of the 43 common interferon pathway genes in tumor (T) and normal (N) tissues of TCGA oral cancer data set.

To systematically explore the altered molecular pathways in oral cancer (Figure 1), the transcriptomic data of TCGA oral cancer (342 tumors vs. 32 normal tissues from USA), GSE23558 (27 tumors vs. 5 normal tissues from India), GSE30784 (167 tumors vs. 45 normal tissues from U.S.A.), GSE37991 (40 paired tumor vs. normal tissues from Taiwan), and GSE138206 (6 paired tumor vs. normal tissues from China) were individually analyzed by GSEA. The significantly upregulated Hallmark pathways of each data sets, which were shown in Supplementary Table S1, were intersected to obtain the 13 common pathways among the 5 oral cancer data sets (Figure 2A). Of note, 6 out of the 13 common Hallmark pathways were related to immune response, including interferon-α response, interferon-γ response, inflammatory response, TNF-α signaling via NF-kB, IL6-JAK-STAT3 signaling, and allograft rejection. Because interferons can modulate both innate and adaptive immune responses, this study first focused on the role of interferon pathways in oral cancer. The enrichment plots demonstrated an obvious activation of interferon-α (Figure 2B) and interferon-γ (Figure 2C) pathways in each of the 5 oral cancer data sets. For each data set, the significant core enriched genes in both interferon pathways were pooled and then intersected to obtain the 43 common interferon pathway genes (Figure 3A), which were upregulated in oral cancer when compared to normal tissues (Figure 3B and Supplementary Figure S1).

### 3.2. The Prognostic Roles of Interferon-Related Genes in Oral Cancer

**Figure 4:**
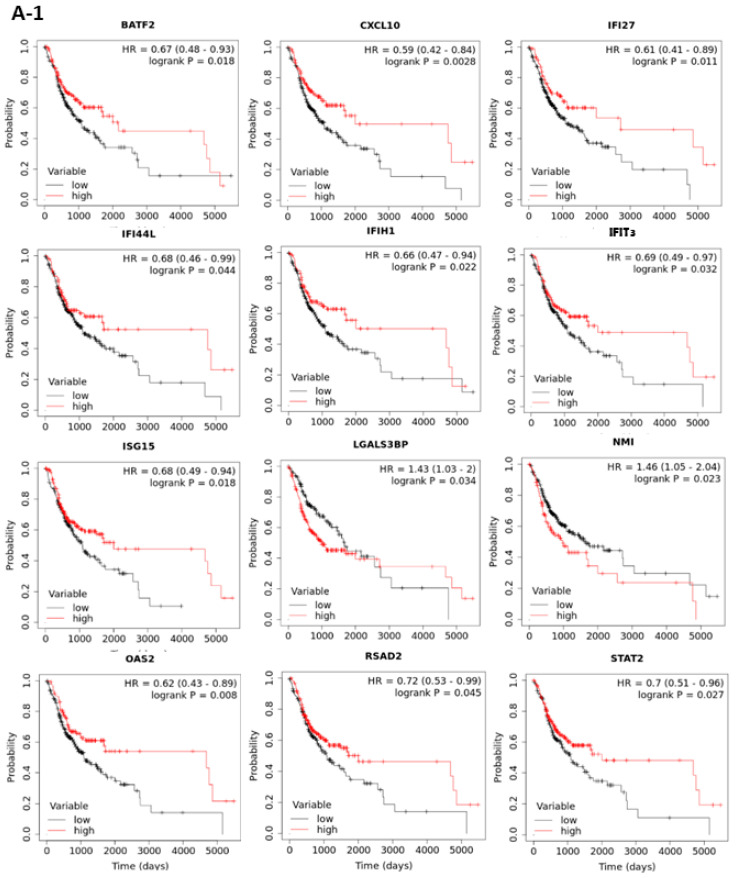

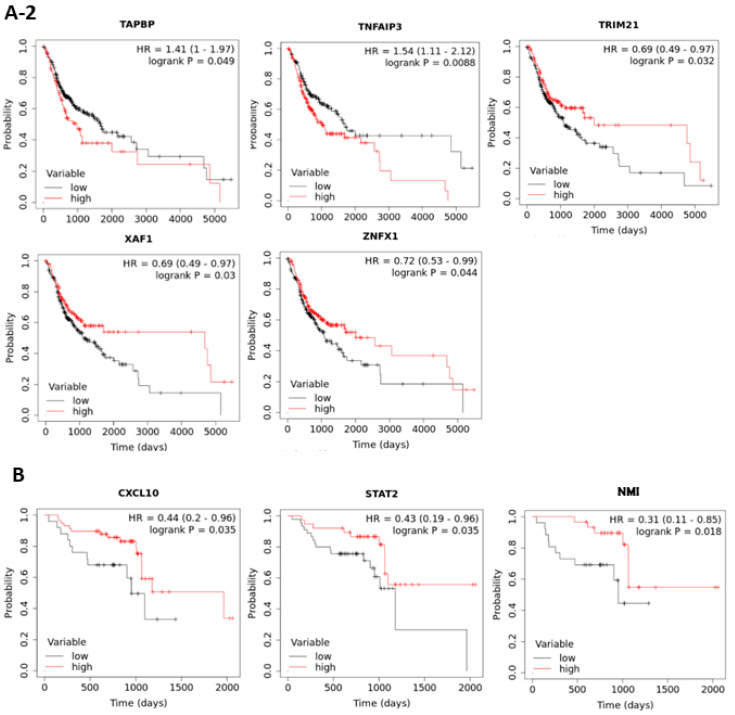
Kaplan-Meier plots of overall survival based on the expressions of interferon pathway genes in TCGA (**A-1**,**A-2**) and GSE65858 (**B**) oral cancer cohorts. The best cut-off values were computed using ROC curve analysis on the Kaplan-Meier plotter (http://kmplot.com/analysis/ accessed between 15 November 2020 and 4 April 2021) online tool. Only the plots with *p* < 0.05 in TCGA (17 out of the 43 genes) and GSE65858 (3 out of the 17 significant genes in TCGA) data sets are shown.

To examine the prognostic roles of the 43 interferon pathway genes in oral cancer, the correlation between patient overall survival (OS) and gene expression in TCGA data set was examined by the web-based tool “Kaplan Meier plotter” (http://kmplot.com/analysis/ accessed between 15 November 2020 and 4 April 2021) with “best cutoff” setting. The results showed that the expressions of 17 genes (BATF2, CXCL10, IFI27, IFI44L, IFIH1, IFIT3, ISG15, LGALS3BP, NMI, OAS2, RSAD2, STAT2, TAPBP, TNFAIP3, TRIM21, XAF1, and ZNFX1) were significantly associated with patient OS (Figure 4(A-1,A-2)). The prognostic values of these 17 interferon pathway genes in oral cancer were validated using another data set (GSE65858) containing 83 oral cancer samples, although some patient characteristics were nonequivalent between TCGA and GSE65858 cohorts (Table 2). The results showed that CXCL10, NMI, and STAT2 were still prognostic in GSE65858 cohort (Figure 4B), the results of the other 14 non-prognostic genes for GSE65858 cohort were shown in Supplementary Figure S2. High expressions of CXCL10 and STAT2 were correlated with favorable patient OS in both cohorts; however, the expression of NMI was inversely associated with patient outcome between TCGA and GSE65858 cohorts (Figure 4(A-1,A-2,B)). Thus, we focused on CXCL10 and STAT2 in the following analyses.

### 3.3. High Expressions of CXCL10 and STAT2 Were Good Prognostic Factors

**Figure 5:**
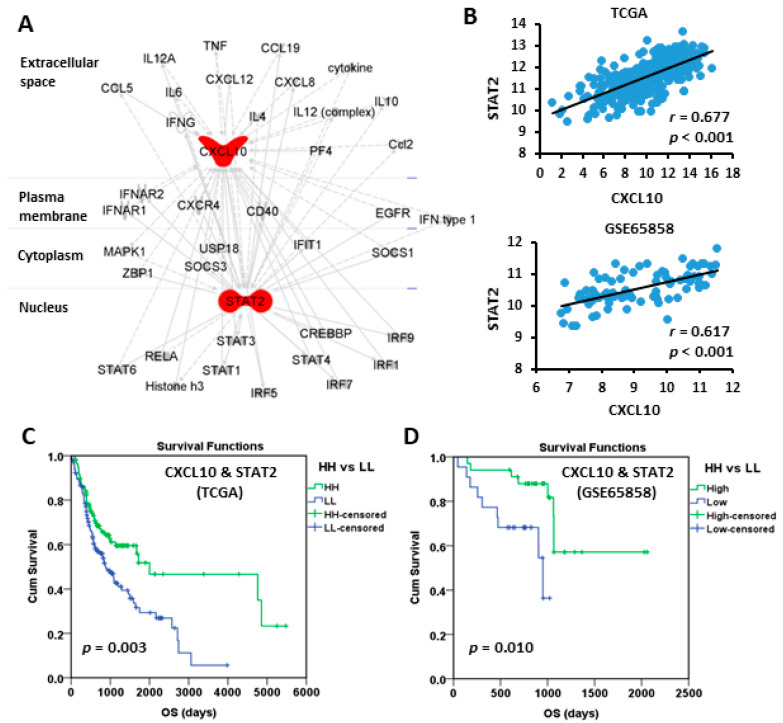
The connection between CXCL10 and STAT2 and the prognostic value based on their coordinated expression. (**A**) Ingenuity Pathway Analysis shows the connection between CXCL10 and STAT2. (**B**) The Spearman correlation between the expressions of CXCL10 and STAT2. (**C**,**D**) Kaplan-Meier plots of overall survival based on the coordinated expression of CXCL10 and STAT2 (HH vs. LL) in TCGA (**C**) and GSE65858 (**D**) oral cancer cohorts. The best cut-off values were computed using ROC curve analysis on the Kaplan-Meier plotter online tool. HH, both high. LL, both low.

The biological correlation between CXCL10 and STAT2 was examined by Path explorer of Ingenuity Pathway Analysis using ingenuity knowledge base, which demonstrated a network connection (Figure 5A). In addition, the expressions of CXCL10 and STAT2 in oral cancer were also correlated in TCGA (*r* = 0.677, *p* < 0.001) and GSE65858 (*r* = 0.617, *p* < 0.001) cohorts (Figure 5B). To take the influence of CXCL10 and STAT2 simultaneously into account of patient outcome, the patients were stratified into “HH” and “LL” subgroups, which represented both high and both low expressions of CXCL10 and STAT2, respectively. The results of Kaplan-Meier survival analysis demonstrated that both the HH patient subgroups in TCGA (Figure 5C) and GSE65858 (Figure 5D) cohorts exhibited good prognosis. The hazard ratio (HR, HH versus LL) was 0.568 (*p* = 0.003) in TCGA and was 0.225 (*p* = 0.010) in GSE65858 in univariate Cox model analysis (Table 3). Large tumor size (T_3–4_) in both cohorts and positive lymph node invasiveness (N_1–3_) in TCGA cohort were significant unfavorable factors for patient OS (Table 3). In multivariate Cox analysis, high CXCL10/STAT2 expression (HH) still showed a protective role in GSE65858 (HR 0.146, *p* = 0.003) but not in TCGA (HR 0.751, *p* = 0.187) cohort (Supplementary Table S2). These results suggest that some confounding factors, such as patient characteristics, may need to be considered for TCGA cohort. Large tumor size (T_3–4_) still increased the risk of death in both cohorts (Supplementary Table S2).

### 3.4. Activation of T-Cell Pathways in the Patients with High CXCL10/STAT2 Expression

**Figure 6:**
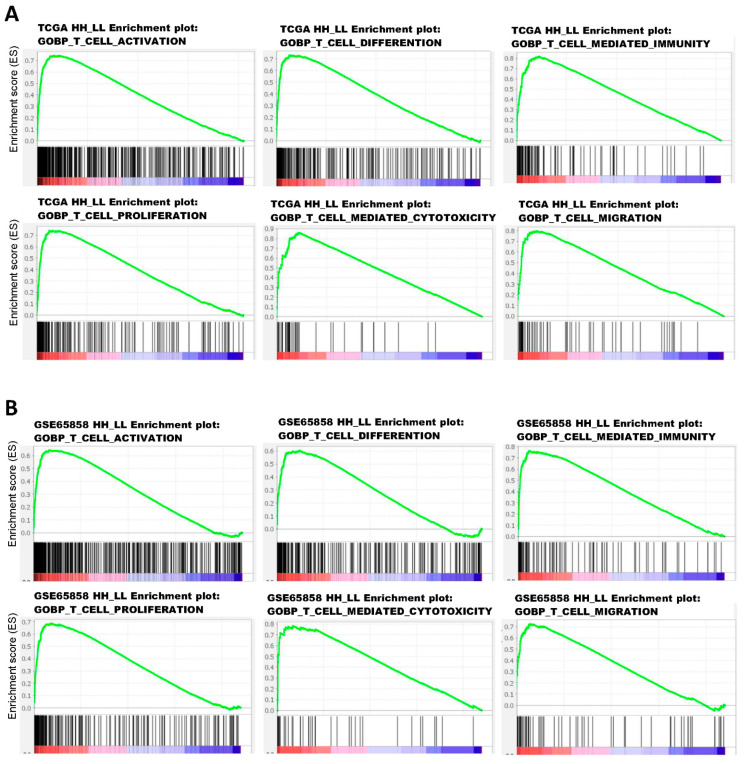
Enrichment plots of GOBP T-cell pathways in TCGA (**A**) and GSE65858 (**B**) oral cancer data sets based on the coordinated expression of CXCL10 and STAT2 (HH vs. LL). The case numbers of HH vs. LL used for GSEA were 108 vs. 144 for TCGA and 34 vs. 22 for GSE65858. GOBP, Gene Ontology Biological Process.

**Figure 7:**
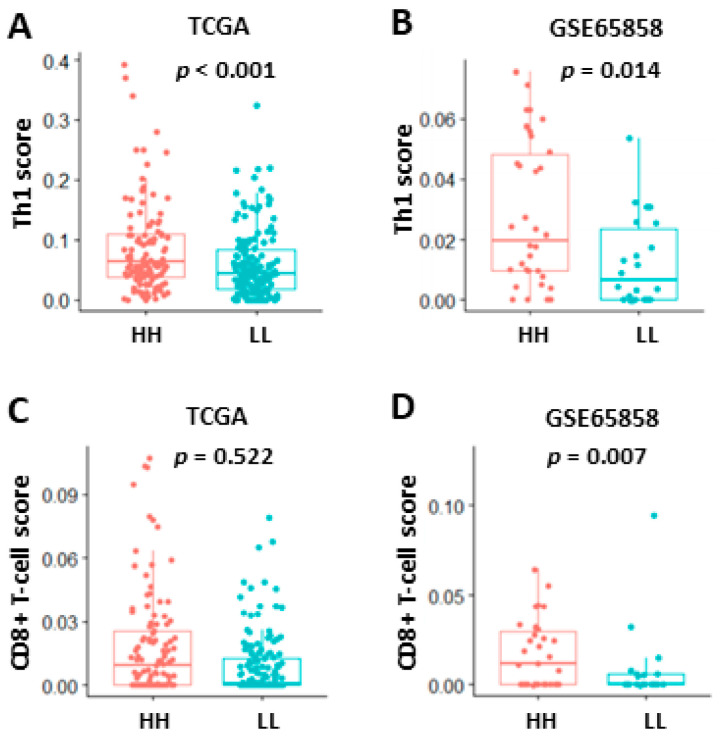
Tumor infiltration of Th1 (**A**,**B**) and CD8+ T cells (**C**,**D**) in TCGA (**A**,**C**) and GSE65858 (**B**,**D**) oral cancer data sets based on the coordinated expression of CXCL10 and STAT2 (HH vs. LL). The analysis was based on xCell algorithm (https://xcell.ucsf.edu/ (accessed on 4 April 2021). HH, both high. LL, both low.

To explore the possible mechanisms underlying the good prognosis in HH patient subgroups, GSEA was performed to compare HH with LL patients in TCGA and GSE65858 cohorts. The results showed that immune-related Hallmark pathways were significantly upregulated in HH patients when compared to LL patients (Table 4). If GSEA was examined using gene sets of Gene Ontology Biological Process (GOBP), upregulation of several T-cell pathways (T-cell activation, differentiation, migration, proliferation, and T-cell-mediated cytotoxicity/immunity) was noted in TCGA (Figure 6A) and GSE65858 (Figure 6B) cohorts. Activation of these T-cell pathways, such as T-cell-mediated immunity, were also observed in GSE23558, GSE30784, GSE37991, and GSE138206 cohorts (Supplementary Figure S3 and data not shown). The tumor infiltration status of T cells in oral cancer was evaluated by xCELL (https://xcell.ucsf.edu/ accessed on 4 April 2021). The results showed that tumor infiltration scores of Th1 cells were significantly higher in HH than in LL patients of TCGA (*p* < 0.001, Figure 7A) and GSE65858 (*p* = 0.014, Figure 7B) cohorts. The scores of CD8+ T-cell were also higher in HH than in LL patients of TCGA (*p* = 0.522, Figure 7C) and GSE65858 (*p* = 0.007, Figure 7D) cohorts, although the difference in TCGA cohort did not reach to statistical significance. These results suggest a close link between high CXCL10/STAT2 expression and T-cell activation, especially for the Th1 and CD8+ T cells, which may contribute to anti-tumor immunity and good patient outcome.

## 4. Discussion

Through comparison of the individual GSEA results from 5 oral cancer data sets (582 tumors and 128 normal samples in total), this study highlights an activation of interferon-α/γ pathways in oral cancer and identifies 43 interferon pathway genes that are commonly upregulated in tumor tissues of the 5 examined data sets. Among the 43 genes, 17 genes are prognostic factors for patient OS in TCGA cohort. The prognostic values of CXCL10 and STAT2 are confirmed in another validation cohort (GSE65858) that contains 83 oral cancer cases. This study also explores the immunological difference between patients with high and low CXCL10/STAT2 expressions (HH and LL subgroups, respectively) by GSEA, which shows activation of T-cell pathways in the HH subgroup. The analysis by xCell algorithm demonstrates that tumors in the HH subgroup exhibit an increased tumor infiltration of Th1 and CD8+ T cells when compared to those in the LL subgroup. These results suggest that high CXCL10/STAT2 expression can predict a favorable outcome in oral cancer patients. The tumor suppressor function of CXCL10 and STAT2 is supported by several lines of evidence. Both CXCL10 and STAT2 are ISGs [11]. In most cells upon type I interferon stimulation, STAT2 is phosphorylated through IFNAR1/IFNAR2-JAK1/TYK2 axis, forming an activated ISGF3 complex, which then translocates into nucleus and binds to the interferon-stimulated response element (ISRE) of CXCL10 promoter to activate its expression [21,22]. CXCL10 is a secretory chemokine that binds to C-X-C motif chemokine receptor 3 (CXCR3), which expresses on CD8+ and CD4+ T cells including Th1 cells. High level of CXCL10 in tumor tissues attracts CXCR3+ Th1 and CD8+ T cells to inflamed tumor sites and stimulates T-cell polarization into effector T cells to promote killing of cancer cells [23]. In a syngeneic murine model of colon cancer, overexpression of CXCL10 suppresses tumor growth in vivo and decreases liver metastasis of colon cancer cells [24]. STAT2 is also required for cytotoxic T-cell response as deletion of Stat2 in antigen-presenting dendritic cells leads to an impairment of cytotoxic T-cell killing in a mouse model [25]. Unlike wild-type mice, interferon-β treatment neither attracts CD4+ and CD8+ T cells to tumor sites nor suppresses tumor growth in Stat2-knockout mice [26]. Furthermore, both CXCL10 and STAT2 are involved in the induction of apoptosis in cancer cells. In vitro studies show that CXCL10 is able to induce apoptosis of cancer cells through upregulating p53 and downregulating Bcl2 [13,27]. Loss of STAT2 in leukemia and osteosarcoma cells leads to resistance of interferon-α-induced apoptosis [14]. These studies clearly indicate the tumor suppressor functions of CXCL10 and STAT2 in cell- and animal-based systems.

The influence of CXCL10 and STAT2 expression on patient survival has been reported in several human cancers. High STAT2 expression is associated with a favorable OS in melanoma and gastric cancer patients [25,28] and is correlated with a better relapse-free survival in breast cancer patients [29]. In contrast, high STAT2 level in glioma predicts poor patient OS [30,31]. Ni et al. have explored the roles of STAT family genes in 520 head and neck cancer patients using the TCGA HNSC dataset but do not find a significant correlation between STAT2 expression and patient OS [32]. Because the TCGA HNSC dataset includes several cancer types other than oral cancer, analysis of the whole TCGA HNSC cohort may result in a missing of clinical significance in some cancer types from different anatomic sites. This study extracts 342 and 83 oral cancer data from TCGA HNSC and GSE65858 (270 head and neck cancers), respectively, and then can demonstrate a significant correlation between good patient OS and high STAT2 expression in both cohorts. This discrepancy suggests that STAT2 is a prognostic biomarker for patients with oral cancer but not with other types of head and neck cancers.

With the exception of osteosarcoma and pancreatic cancer [33,34,35], high CXCL10 expression is associated with favorable patient outcomes in various cancer types including breast, ovarian, liver, colon, rectum, esophagus, thyroid, and head and neck cancers [36,37,38,39,40,41,42,43]. Different from the aforementioned case of STAT2, high CXCL10 expression still predicts good prognosis in the whole TCGA HNSC cohort [43]. The beneficial effect of high CXCL10 expression on patient outcome is, at least in part, correlated with the chemotaxis activity of CXCL10. In thyroid cancer, high CXCL10 expression is associated with increased tumor infiltration of immune cells including CD8+ T cells, which predicts better patient OS [42]. This study also demonstrates that high CXCL10/STAT2 expression (HH subgroup) is associated with activation of T-cell pathways, increased tumor infiltration of Th1 and CD8+ T cells, and good patient prognosis. In addition to TCGA and GSE65858 cohorts, activation of T-cell pathways is observed in another 4 oral cancer data sets examined in this study (Supplementary Figure S3 and data not shown). Although the clinical information is not publicly available for these 4 oral cancer data sets, it is possible that the patients with high CXCL10/STAT2 expression also have a more favorable outcome than those with low CXCL10/STAT2 expression.

Korpela et al. report that anti-EGFR therapeutics can induce the expression of CXCL10, STAT2, and interferon responses in human head and neck cancer cell lines as well as in a murine model [44]. They also find an increased tumor infiltration of CD8+ T cells in head and neck cancer patients who receive and respond to cetuximab, an anti-EGFR therapeutic antibody [44]. Furthermore, several recent studies have demonstrated that high CXCL10 level is an indicator of positive response to immune checkpoint blockade (ICB) in melanoma, neuroblastoma, breast and urothelial cancers [45,46,47,48]. These results suggest that high CXCL10/STAT2 expression may be a biomarker for selection of oral cancer patients to receive anti-EGFR and/or immunotherapy. It is worth specifically examining the biomarker role of CXCL10/STAT2 in responses to anti-EGFR and ICB-based therapies in oral cancer patients.

There are still some concerning issues and limitation in the present study. Rentoft et al. report a contradictory result in 38 patients with tongue cancer who received neoadjuvant radiotherapy. They find that patients with high CXCL10 expression exhibit poor OS [49]. Therefore, it is required to discriminate the differential effects of CXCL10 and radiotherapy on patient outcome before and after surgery. In another study [50], Li et al. compare gene expression between 56 oral cancer samples and 56 normal tissues from 7 data sets (among these only GSE30784 is included in this study) and find that the interferon pathway genes CXCL10 and IFI27 are upregulated in oral cancer, which results are the same as the present study. However, Li et al. do not examine the prognostic roles of CXCL10 and IFI27 due to lack of clinical data for the 7 data sets. Instead, they conduct a cell-based study and find that knockdown of CXCL10 in oral cancer cells leads to reduction of cell proliferation, migration and invasion, which suggests an oncogenic role of CXCL10 in this in vitro cell model. Thus, the cell autonomous and non-autonomous functions of CXCL10 in the tumor microenvironment of oral cancer may need to be further investigated. In the present study, there are missing data of T- and N-stages in multivariate Cox analysis of the TCGA data set, which may affect the significance of statistical results (Supplementary Table S2). We also note that several patient characteristics, such as age, T-stage, smoking and survival status are different between TCGA and GSE65858 cohorts (Table 2), which may influence the seeking of common prognostic genes (only CXCL10 and STAT2) among the two cohorts. For this reason, we cannot simply exclude the prognostic values of another 15 interferon pathway genes that are identified in the TCGA cohort. In the future, it will be necessary to include more patient data to validate the roles of these interferon pathway genes in oral cancer.

## 5. Conclusions

Upregulation of interferon pathway genes are observed in many oral cancer data sets. High expressions of CXCL10 and STAT2, which are correlated with activation of T-cell pathways and tumor infiltration of Th1 and CD8+ T cells, are good prognostic biomarkers for oral cancer patients. 

## Figures and Tables

**Table 1:** The 5 oral cancer data sets used for gene set enrichment analysis.

Cohort		TCGA ^1^ (USA)	GSE23558 (India)	GSE30784 (USA)	GSE37991 (Taiwan)	GSE138206 (China)
Case number	Tumor	342	27	167	40 ^2^	6 ^2^
	Normal	32	5	45	40	6
Gender (male/female)	Tumor	236/106	20/7	120/47	40/0	5/1
	Normal	20/12	3/1 ^3^	32/13	40/0	5/1
Age (yr)	Tumor	19–90	31–78	19–88	-	-
	Normal	29–87	25–56	19–88	-	-

^1^ Only oral cavity cancer. ^2^ Tumor-normal pair. ^3^ One normal control is a pool of 5 male and 4 female samples.

**Table 2:** Patient demographics of TCGA and GSE65858 cohorts ^1^.

		TCGA	GSE65858	*p*-Value
	Total case No.	342	83	
Gender	Male	236 (69.0%)	64 (77.1%)	0.146
	Female	106 (31.0%)	19 (22.9%)	
Age (yr)	median (range)	61 (19–90)	57 (35–87)	
	>60	187 (54.7%)	36 (43.4%)	0.042
	<60	148 (43.3%)	47 (56.6%)	
T stage	T1	34 (9.9%)	14 (16.9%)	0.022
	T2	100 (29.2%)	22 (26.5%)	
	T3	68 (19.9%)	8 (9.6%)	
	T4	113 (33.0%)	39 (47.0%)	
N stage	N0	123 (36.0%)	36 (43.4%)	0.352
	N1	51 (14.9%)	9 (10.8%)	
	N2	109 (31.9%)	36 (43.4%)	
	N3	3 (0.9%)	2 (2.4%)	
Status	Alive	188 (55.0%)	56 (67.5%)	0.039
	Dead	154 (45.0%)	27 (32.5%)	
OS ^2^ (day)	median (range)	641 (11–5480)	831 (46–2059)	
Smoking	Yes	180 (52.6%)	66 (79.5%)	<0.001
	No	162 (47.4%)	17 (20.5%)	

^1^ Only oral cavity cancer, all are squamous cell carcinoma. ^2^ Overall survival.

**Table 3:** Univariate Cox model analysis of overall survival.

TCGA	Case No.	HR	95% CI	*p*
HH vs. LL ^1^	108 vs. 144	0.568	0.389–0.828	0.003
T_3–4_ vs. T_1–2_	141 vs. 97	2.448	1.602–3.740	<0.001
N_1–3_ vs. N_0_	126 vs. 97	1.776	1.187–2.657	0.005
Male vs. Female	177 vs. 75	1.146	0.779–1.685	0.189
>60-yr vs. <60-yr	147 vs. 105	1.159	0.808–1.163	0.423
**GSE65858**	**Case No.**	**HR**	**95% CI**	***p***
HH vs. LL ^1^	34 vs. 22	0.225	0.072–0.705	0.010
T_3–4_ vs. T_1–2_	29 vs. 27	4.985	1.428–17.397	0.012
N_1–3_ vs. N_0_	31 vs. 25	2.271	0.798–6.467	0.124
Male vs. Female	42 vs. 14	0.743	0.274–2.017	0.560
>60-yr vs. <60-yr	25 vs. 31	0.747	0.272–2.054	0.572

^1^ CXCL10/STAT2 expression, both high (HH), both low (LL).

**Table 4:** Significantly upregulated immune-related Hallmark pathways in HH subgroup.

TCGA (HH vs. LL) ^1^	SIZE	ES	NES	NOM-p	FDR-q
HALLMARK_INTERFERON_GAMMA_RESPONSE	196	0.8661	3.2608	0.0000	0.0000
HALLMARK_ALLOGRAFT_REJECTION	195	0.8450	3.1759	0.0000	0.0000
HALLMARK_INTERFERON_ALPHA_RESPONSE	92	0.8889	3.0438	0.0000	0.0000
HALLMARK_INFLAMMATORY_RESPONSE	197	0.7469	2.8229	0.0000	0.0000
HALLMARK_IL6_JAK_STAT3_SIGNALING	87	0.7571	2.5515	0.0000	0.0000
HALLMARK_COMPLEMENT	195	0.6451	2.4374	0.0000	0.0000
HALLMARK_TNFA_SIGNALING_VIA_NFKB	197	0.6280	2.3792	0.0000	0.0000
HALLMARK_IL2_STAT5_SIGNALING	194	0.6107	2.2753	0.0000	0.0000
**GSE65858 (HH vs. LL) ^1^**	**SIZE**	**ES**	**NES**	**NOM-p**	**FDR-q**
HALLMARK_INTERFERON_GAMMA_RESPONSE	194	0.8920	3.0061	0.0000	0.0000
HALLMARK_INTERFERON_ALPHA_RESPONSE	91	0.9404	2.8835	0.0000	0.0000
HALLMARK_ALLOGRAFT_REJECTION	178	0.8150	2.7355	0.0000	0.0000
HALLMARK_INFLAMMATORY_RESPONSE	189	0.7444	2.5251	0.0000	0.0000
HALLMARK_COMPLEMENT	183	0.7261	2.4306	0.0000	0.0000
HALLMARK_IL6_JAK_STAT3_SIGNALING	82	0.7646	2.3251	0.0000	0.0000
HALLMARK_TNFA_SIGNALING_VIA_NFKB	192	0.6421	2.1593	0.0000	0.0000
HALLMARK_IL2_STAT5_SIGNALING	184	0.5779	1.9426	0.0000	0.0000

^1^ CXCL10/STAT2 expression, both high (HH), both low (LL).

## Data Availability

All data supporting reported results can be found through requesting the corresponding author. The TCGA and GEO oral cancer data sets can be accessed at https://portal.gdc.cancer.gov and https://www.ncbi.nlm.nih.gov/geo/ (data accessed on 15 July 2020), respectively.

## Supplementary Materials

The following are available online at https://www.mdpi.com/article/10.3390/jpm12020140/s1, Figure S1: Heat map showing the differential expression of the 43 common interferon pathway genes in tumor (T) and normal (N) tissues of GSE23558 (A), GSE30784 (B), GSE37991 (C), and GSE138206 (D) data sets, Figure S2: Kaplan-Meier plots of overall survival based on the 14 interferon pathway genes in GSE65858 data set, Figure S3: Enrichment plots of GOBP T-cell-mediated immunity pathway in GSE23558, GSE20784, GSE37991, and GSE138206 data sets based on the coordinated expression of CXCL10 and STAT2 (HH vs. LL), Table S1: Significant enriched Hallmark pathways in each of the 5 oral cancer data sets, Table S2: Multivariate Cox model analysis of overall survival.
