# Supplementary material for: High Expression of Interferon Pathway Genes CXCL10 and STAT2 Is Associated with Activated T-Cell Signature and Better Outcome of Oral Cancer Patients
Source: J Pers Med. 2022 Jan 21;12(2):140. doi: 10.3390/jpm12020140 (PMC8877377; doi:10.3390/jpm12020140)
Supplement: Supplementary file 1 [file jpm-12-00140-s001.zip › jpm-1533367-supplementary.pdf]

Supplementary Materials

High expression of interferon pathway genes CXCL10 and STAT2 is correlated with activation of T-cell signature and better outcome of oral cancer patients

Yun-Cian Huang [1], Jau-Ling Huang [2], Lu-Chia Tseng [2], Ping-Hung Yu [3], Si-Yun Chen [1] and Chang-Shen Lin [1,4,5,6,*]

[1] Graduate Institute of Medicine, College of Medicine, Kaohsiung Medical University, Kaohsiung 807, Taiwan; yuncian1103@gmail.com (Y.C.H.); qoxoruby@gmail.com (S.Y.C.); csl@kmu.edu.tw (C.S.L.)
[2] Department of Bioscience Technology, College of Health Science, Chang Jung Christian University, Tainan 711, Taiwan; jaulingh@mail.cjcu.edu.tw (J.L.H.); luke20000820@gmail.com (L.C.T.)
[3] Department of Nursing, National Taichung University of Science and Technology, Taichung 404, Taiwan; pt1013@nutc.edu.tw; oxfordocean@gmail.com (P.H.Y.)
[4] Center for Cancer Research, Kaohsiung Medical University, Kaohsiung, Taiwan
[5] Department of Medical Research, Kaohsiung Medical University Hospital, Kaohsiung Medical University, Kaohsiung, Taiwan
[6] Department of Biological Sciences, National Sun Yat-sen University, Kaohsiung, Taiwan
***** Correspondence: csl@kmu.edu.tw; changshenlin@gmail.com

Table S1. Significant enriched Hallmark pathways in each of the 5 oral cancer data sets. (Corresponding to Figure 2).

**TCGA**	**ES**	**NES**	**NOM-p**	**FDR-q**
HALLMARK_E2F_TARGETS	0.68	2.89	0.00	0.00
HALLMARK_G2M_CHECKPOINT	0.66	2.77	0.00	0.00
HALLMARK_INTERFERON_ALPHA_RESPONSE	0.66	2.53	0.00	0.00
HALLMARK_EPITHELIAL_MESENCHYMAL_TRANSITION	0.58	2.46	0.00	0.00
HALLMARK_INTERFERON_GAMMA_RESPONSE	0.54	2.31	0.00	0.00
HALLMARK_MITOTIC_SPINDLE	0.51	2.14	0.00	0.00
HALLMARK_ANGIOGENESIS	0.55	1.83	0.00	0.00
HALLMARK_MYC_TARGETS_V1	0.41	1.73	0.00	0.00
HALLMARK_INFLAMMATORY_RESPONSE	0.40	1.71	0.00	0.00
HALLMARK_ALLOGRAFT_REJECTION	0.40	1.69	0.00	0.00
HALLMARK_DNA_REPAIR	0.38	1.57	0.00	0.01
HALLMARK_MYC_TARGETS_V2	0.44	1.54	0.01	0.01
HALLMARK_TNFA_SIGNALING_VIA_NFKB	0.36	1.52	0.00	0.02
HALLMARK_MTORC1_SIGNALING	0.35	1.45	0.00	0.03
HALLMARK_IL6_JAK_STAT3_SIGNALING	0.37	1.38	0.02	0.05
HALLMARK_GLYCOLYSIS	0.33	1.37	0.00	0.05
HALLMARK_APICAL_JUNCTION	0.31	1.34	0.01	0.06
HALLMARK_COAGULATION	0.32	1.30	0.03	0.08
**GSE23558**	**ES**	**NES**	**NOM-p**	**FDR-q**
HALLMARK_E2F_TARGETS	0.71	3.45	0.00	0.00
HALLMARK_INTERFERON_ALPHA_RESPONSE	0.73	3.18	0.00	0.00
HALLMARK_INTERFERON_GAMMA_RESPONSE	0.62	2.99	0.00	0.00
HALLMARK_G2M_CHECKPOINT	0.61	2.93	0.00	0.00
HALLMARK_MYC_TARGETS_V2	0.70	2.80	0.00	0.00
HALLMARK_MYC_TARGETS_V1	0.57	2.73	0.00	0.00
HALLMARK_TNFA_SIGNALING_VIA_NFKB	0.52	2.54	0.00	0.00
HALLMARK_MTORC1_SIGNALING	0.48	2.31	0.00	0.00
HALLMARK_DNA_REPAIR	0.49	2.26	0.00	0.00
HALLMARK_MITOTIC_SPINDLE	0.45	2.14	0.00	0.00
HALLMARK_IL6_JAK_STAT3_SIGNALING	0.47	2.04	0.00	0.00
HALLMARK_UNFOLDED_PROTEIN_RESPONSE	0.42	1.86	0.00	0.00
HALLMARK_GLYCOLYSIS	0.37	1.78	0.00	0.00
HALLMARK_INFLAMMATORY_RESPONSE	0.36	1.78	0.00	0.00
HALLMARK_APICAL_JUNCTION	0.34	1.66	0.00	0.01
HALLMARK_UV_RESPONSE_UP	0.33	1.55	0.00	0.01
HALLMARK_EPITHELIAL_MESENCHYMAL_TRANSITION	0.31	1.52	0.00	0.01
HALLMARK_P53_PATHWAY	0.31	1.49	0.00	0.02
HALLMARK_APOPTOSIS	0.31	1.45	0.00	0.02
HALLMARK_COMPLEMENT	0.30	1.44	0.00	0.02
HALLMARK_ALLOGRAFT_REJECTION	0.30	1.44	0.00	0.02
HALLMARK_ANGIOGENESIS	0.40	1.41	0.05	0.03
HALLMARK_KRAS_SIGNALING_UP	0.29	1.39	0.01	0.03
HALLMARK_IL2_STAT5_SIGNALING	0.28	1.37	0.01	0.04
HALLMARK_OXIDATIVE_PHOSPHORYLATION	0.27	1.27	0.03	0.08

GSE30784	ES	NES	NOM-p	FDR-q
HALLMARK_EPITHELIAL_MESENCHYMAL_TRANSITION	0.79	2.49	0.00	0.00
HALLMARK_INTERFERON_ALPHA_RESPONSE	0.80	2.33	0.00	0.00
HALLMARK_INTERFERON_GAMMA_RESPONSE	0.72	2.28	0.00	0.00
HALLMARK_TNFA_SIGNALING_VIA_NFKB	0.72	2.27	0.00	0.00
HALLMARK_INFLAMMATORY_RESPONSE	0.71	2.24	0.00	0.00
HALLMARK_ALLOGRAFT_REJECTION	0.66	2.09	0.00	0.00
HALLMARK_IL6_JAK_STAT3_SIGNALING	0.72	2.07	0.00	0.00
HALLMARK_E2F_TARGETS	0.65	2.06	0.00	0.00
HALLMARK_ANGIOGENESIS	0.82	2.05	0.00	0.00
HALLMARK_TGF_BETA_SIGNALING	0.72	1.90	0.00	0.00
HALLMARK_G2M_CHECKPOINT	0.60	1.87	0.00	0.00
HALLMARK_HEDGEHOG_SIGNALING	0.74	1.85	0.00	0.00
HALLMARK_APOPTOSIS	0.57	1.76	0.00	0.00
HALLMARK_COMPLEMENT	0.55	1.73	0.00	0.00
HALLMARK_UV_RESPONSE_UP	0.54	1.67	0.00	0.00
HALLMARK_COAGULATION	0.54	1.65	0.00	0.00
HALLMARK_KRAS_SIGNALING_UP	0.52	1.63	0.00	0.00
HALLMARK_HYPOXIA	0.52	1.63	0.00	0.00
HALLMARK_UV_RESPONSE_DN	0.53	1.62	0.00	0.00
HALLMARK_MITOTIC_SPINDLE	0.50	1.55	0.00	0.00
HALLMARK_APICAL_JUNCTION	0.48	1.53	0.00	0.01
HALLMARK_UNFOLDED_PROTEIN_RESPONSE	0.52	1.52	0.00	0.01
HALLMARK_MTORC1_SIGNALING	0.47	1.47	0.00	0.01
HALLMARK_IL2_STAT5_SIGNALING	0.49	1.53	0.00	0.01
HALLMARK_MYC_TARGETS_V2	0.63	1.67	0.00	0.01
HALLMARK_GLYCOLYSIS	0.44	1.40	0.00	0.02
HALLMARK_MYC_TARGETS_V1	0.42	1.34	0.02	0.04
HALLMARK_DNA_REPAIR	0.43	1.31	0.03	0.05
**GSE138206**	**ES**	**NES**	**NOM-p**	**FDR-q**
HALLMARK_INTERFERON_ALPHA_RESPONSE	0.66	3.07	0.00	0.00
HALLMARK_EPITHELIAL_MESENCHYMAL_TRANSITION	0.56	3.02	0.00	0.00
HALLMARK_INTERFERON_GAMMA_RESPONSE	0.56	3.01	0.00	0.00
HALLMARK_ALLOGRAFT_REJECTION	0.52	2.67	0.00	0.00
HALLMARK_E2F_TARGETS	0.51	2.55	0.00	0.00
HALLMARK_IL6_JAK_STAT3_SIGNALING	0.53	2.38	0.00	0.00
HALLMARK_INFLAMMATORY_RESPONSE	0.45	2.37	0.00	0.00
HALLMARK_ANGIOGENESIS	0.57	2.12	0.00	0.00
HALLMARK_COMPLEMENT	0.39	1.97	0.00	0.00
HALLMARK_TGF_BETA_SIGNALING	0.49	1.90	0.00	0.00
HALLMARK_TNFA_SIGNALING_VIA_NFKB	0.35	1.84	0.00	0.00
HALLMARK_G2M_CHECKPOINT	0.37	1.83	0.00	0.00
HALLMARK_DNA_REPAIR	0.43	1.78	0.00	0.00
HALLMARK_APICAL_JUNCTION	0.33	1.63	0.00	0.01
HALLMARK_COAGULATION	0.34	1.58	0.01	0.02
HALLMARK_UNFOLDED_PROTEIN_RESPONSE	0.40	1.55	0.03	0.02
HALLMARK_KRAS_SIGNALING_UP	0.28	1.46	0.00	0.04
HALLMARK_MITOTIC_SPINDLE	0.29	1.43	0.02	0.06
HALLMARK_APOPTOSIS	0.28	1.40	0.02	0.06

**GSE37991**	**ES**	**NES**	**NOM-p**	**FDR-q**
HALLMARK_INTERFERON_ALPHA_RESPONSE	0.69	2.74	0.00	0.00
HALLMARK_E2F_TARGETS	0.57	2.54	0.00	0.00
HALLMARK_INTERFERON_GAMMA_RESPONSE	0.55	2.51	0.00	0.00
HALLMARK_G2M_CHECKPOINT	0.53	2.33	0.00	0.00
HALLMARK_EPITHELIAL_MESENCHYMAL_TRANSITION	0.51	2.30	0.00	0.00
HALLMARK_MITOTIC_SPINDLE	0.43	1.95	0.00	0.00
HALLMARK_MYC_TARGETS_V2	0.54	1.90	0.00	0.00
HALLMARK_DNA_REPAIR	0.43	1.84	0.00	0.00
HALLMARK_UNFOLDED_PROTEIN_RESPONSE	0.45	1.82	0.00	0.00
HALLMARK_INFLAMMATORY_RESPONSE	0.40	1.82	0.00	0.00
HALLMARK_ALLOGRAFT_REJECTION	0.40	1.80	0.00	0.00
HALLMARK_COMPLEMENT	0.40	1.80	0.00	0.00
HALLMARK_MYC_TARGETS_V1	0.40	1.78	0.00	0.00
HALLMARK_APICAL_JUNCTION	0.39	1.77	0.00	0.00
HALLMARK_MTORC1_SIGNALING	0.38	1.72	0.00	0.00
HALLMARK_APOPTOSIS	0.38	1.70	0.00	0.00
HALLMARK_TNFA_SIGNALING_VIA_NFKB	0.37	1.69	0.00	0.00
HALLMARK_TGF_BETA_SIGNALING	0.46	1.67	0.00	0.00
HALLMARK_IL6_JAK_STAT3_SIGNALING	0.39	1.58	0.00	0.01
HALLMARK_ANGIOGENESIS	0.46	1.54	0.02	0.01
HALLMARK_GLYCOLYSIS	0.33	1.52	0.00	0.01
HALLMARK_IL2_STAT5_SIGNALING	0.31	1.38	0.01	0.04
HALLMARK_COAGULATION	0.31	1.36	0.02	0.05
HALLMARK_KRAS_SIGNALING_UP	0.30	1.35	0.02	0.05
HALLMARK_P53_PATHWAY	0.28	1.29	0.02	0.08

Table S2. Multivariate Cox model analysis of overall survival.

TCGA	Case No.	HR	95% CI	*p*
HH vs LL[a]	91 vs 128	0.751	0.491-1.149	0.187
$T_{3\text{-}4}$ vs $T_{1\text{-}2}$	137 vs 82	2.384	1.465-3.878	<0.001
$N_{1\text{-}3}$ vs N_0	125 vs 94	1.463	0.966-2.215	0.072
**GSE65858**	**Case No.**	**HR**	**95% CI**	***p***
HH vs LL[a]	34 vs 22	0.146	0.041-0.522	0.003
$T_{3\text{-}4}$ vs $T_{1\text{-}2}$	29 vs 27	6.912	1.872-25.520	0.004

[a] CXCL10 and STAT2 expression, both high (HH), both low (LL).

A

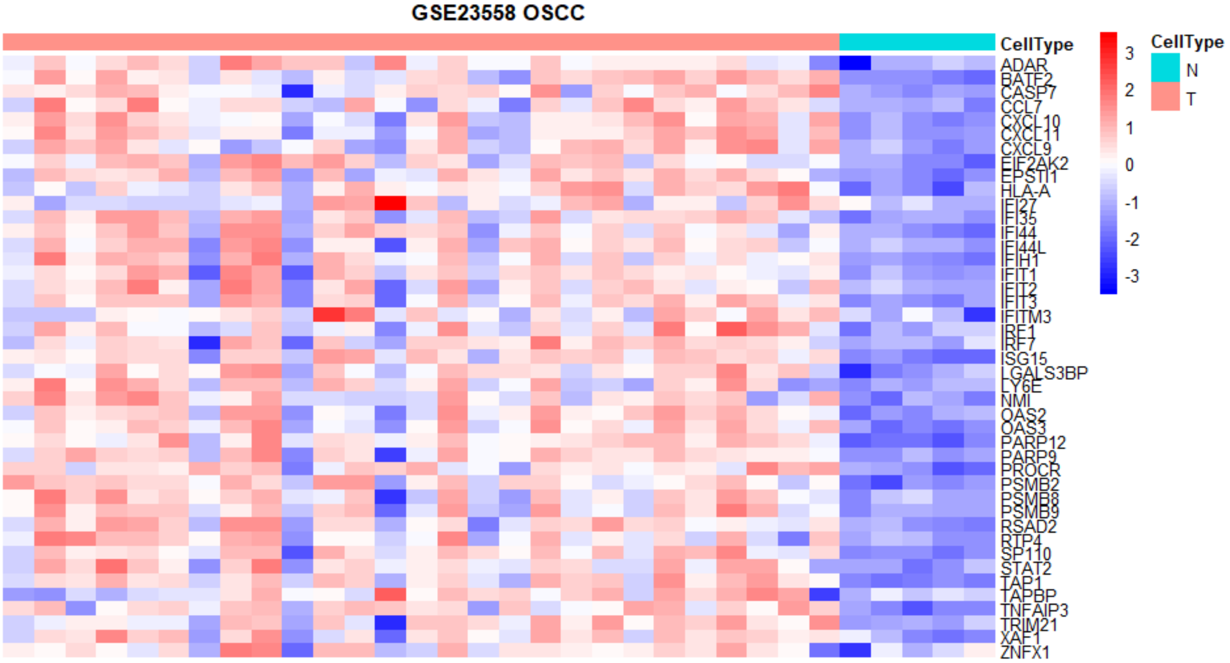
GSE23558 OSCC
CellType
ADAR
BATF2
CASP7
CCL7
CXCL10
CXCL11
CXCL9
EIF2AK2
EPSTI1
HLA-A
IFI27
IFI35
IFI44
IFI44L
IFIH1
IFIT1
IFIT2
IFIT3
IFITM3
IRF1
IRF7
ISG15
LGALS3BP
LY6E
NMI
OAS2
OAS3
PARP12
PARP9
PROCR
PSMB2
PSMB8
PSMB9
RSAD2
RTP4
SP110
STAT2
TAP1
TAPBP
TNFAIP3
TRIM21
XAF1
ZNFX1
3
2
1
0
-1
-2
-3
CellType
N
T

B

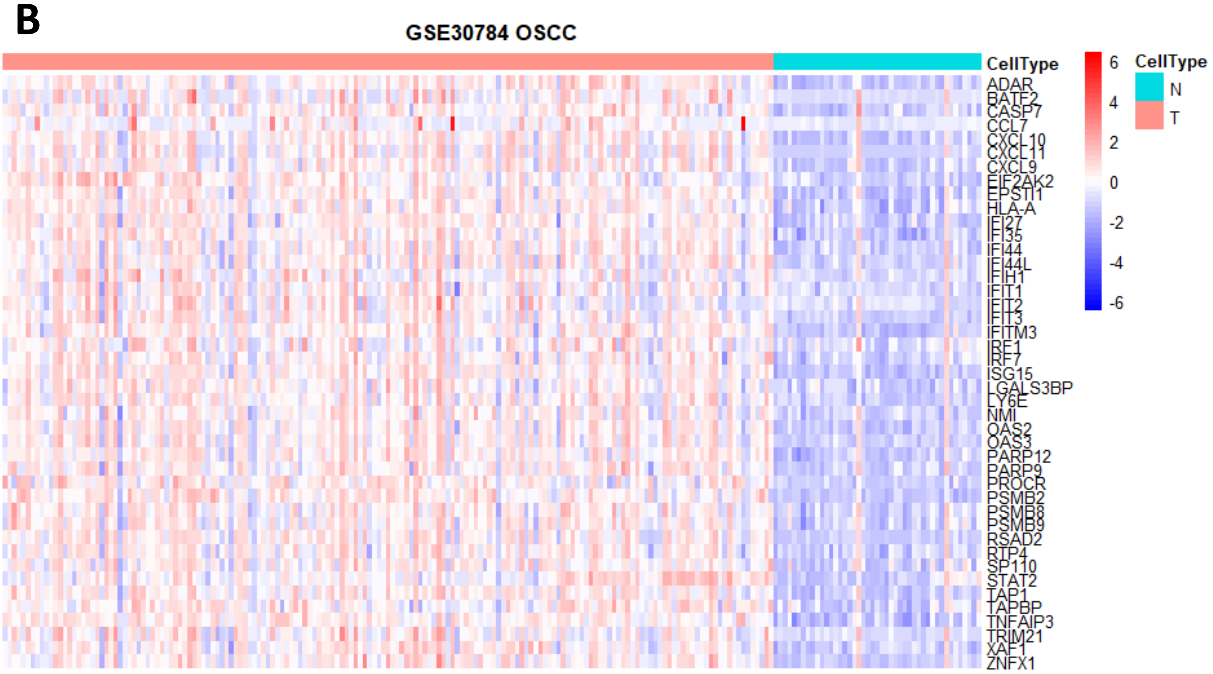
GSE30784 OSCC
CellType
ADAR
BATF2
CASP7
CCL7
CXCL10
CXCL11
CXCL9
EIF2AK2
EPSTI1
HLA-A
IFI27
IFI35
IFI44
IFI44L
IFIH1
IFIT1
IFIT2
IFIT3
IFITM3
IRF1
IRF7
ISG15
LGALS3BP
LY6E
NMI
OAS2
OAS3
PARP12
PARP9
PROCR
PSMB2
PSMB8
PSMB9
RSAD2
RTP4
SP110
STAT2
TAP1
TAPBP
TNFAIP3
TRIM21
XAF1
ZNFX1
6
4
2
0
-2
-4
-6
CellType
N
T

C

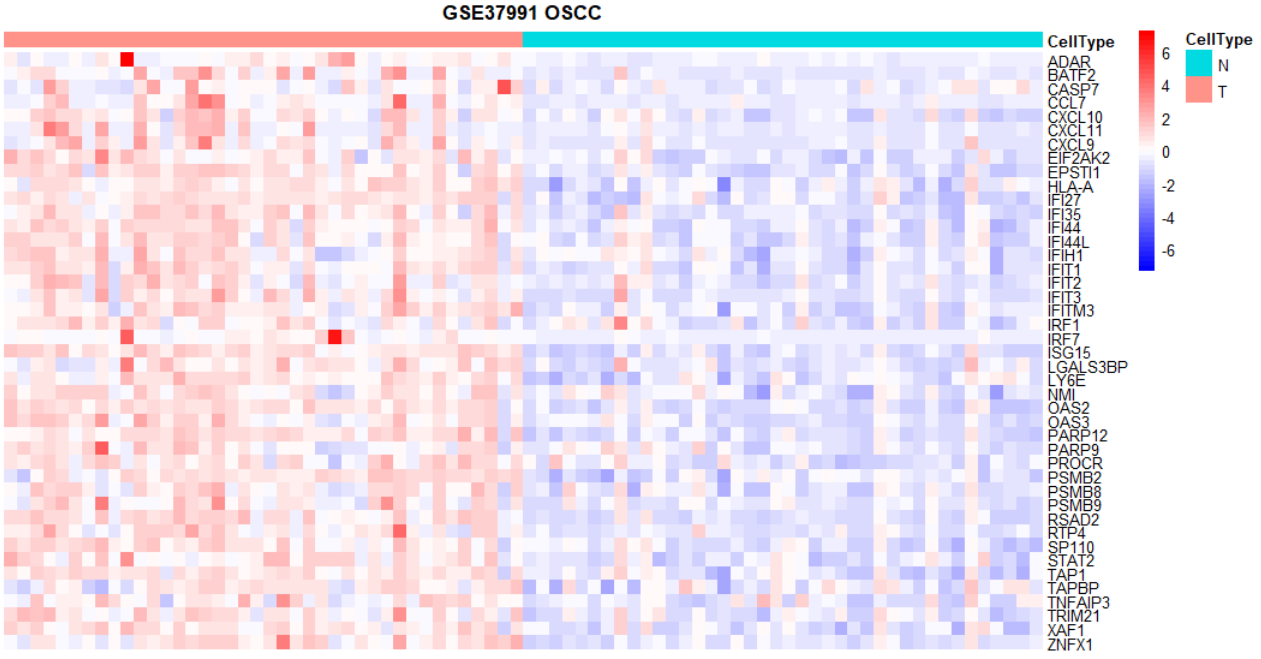

D

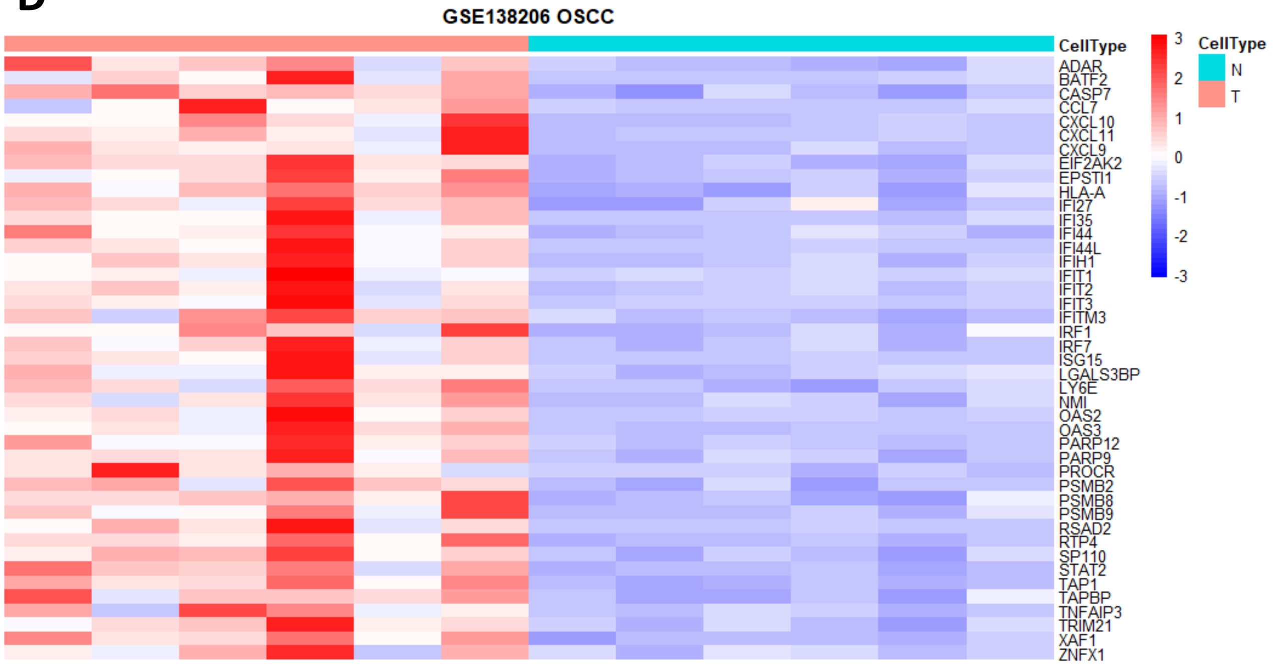

Figure S1. Heat map showing the differential expression of the 43 common interferon pathway genes in tumor (T) and normal (N) tissues of GSE23558 (A), GSE30784 (B), GSE37991 (C), and GSE138206 (D) data sets.(Corresponding to Figure 3).

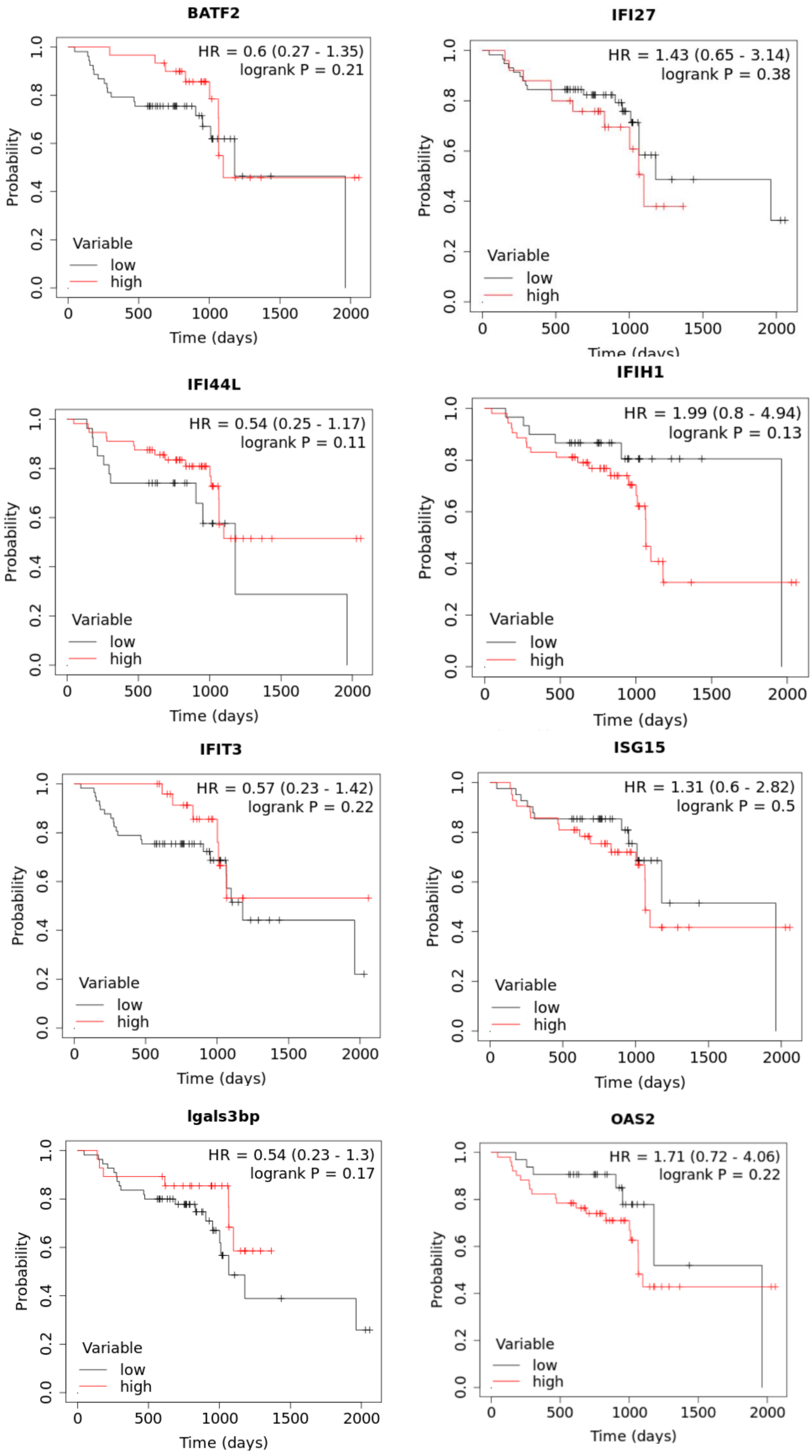
BATF2
HR = 0.6 (0.27 - 1.35)
logrank P = 0.21
Probability
Time (days)
Variable
low
high
IFI27
HR = 1.43 (0.65 - 3.14)
logrank P = 0.38
Probability
Time (days)
Variable
low
high
IFI44L
HR = 0.54 (0.25 - 1.17)
logrank P = 0.11
Probability
Time (days)
Variable
low
high
IFIH1
HR = 1.99 (0.8 - 4.94)
logrank P = 0.13
Probability
Time (days)
Variable
low
high
IFIT3
HR = 0.57 (0.23 - 1.42)
logrank P = 0.22
Probability
Time (days)
Variable
low
high
ISG15
HR = 1.31 (0.6 - 2.82)
logrank P = 0.5
Probability
Time (days)
Variable
low
high
lgals3bp
HR = 0.54 (0.23 - 1.3)
logrank P = 0.17
Probability
Time (days)
Variable
low
high
OAS2
HR = 1.71 (0.72 - 4.06)
logrank P = 0.22
Probability
Time (days)
Variable
low
high

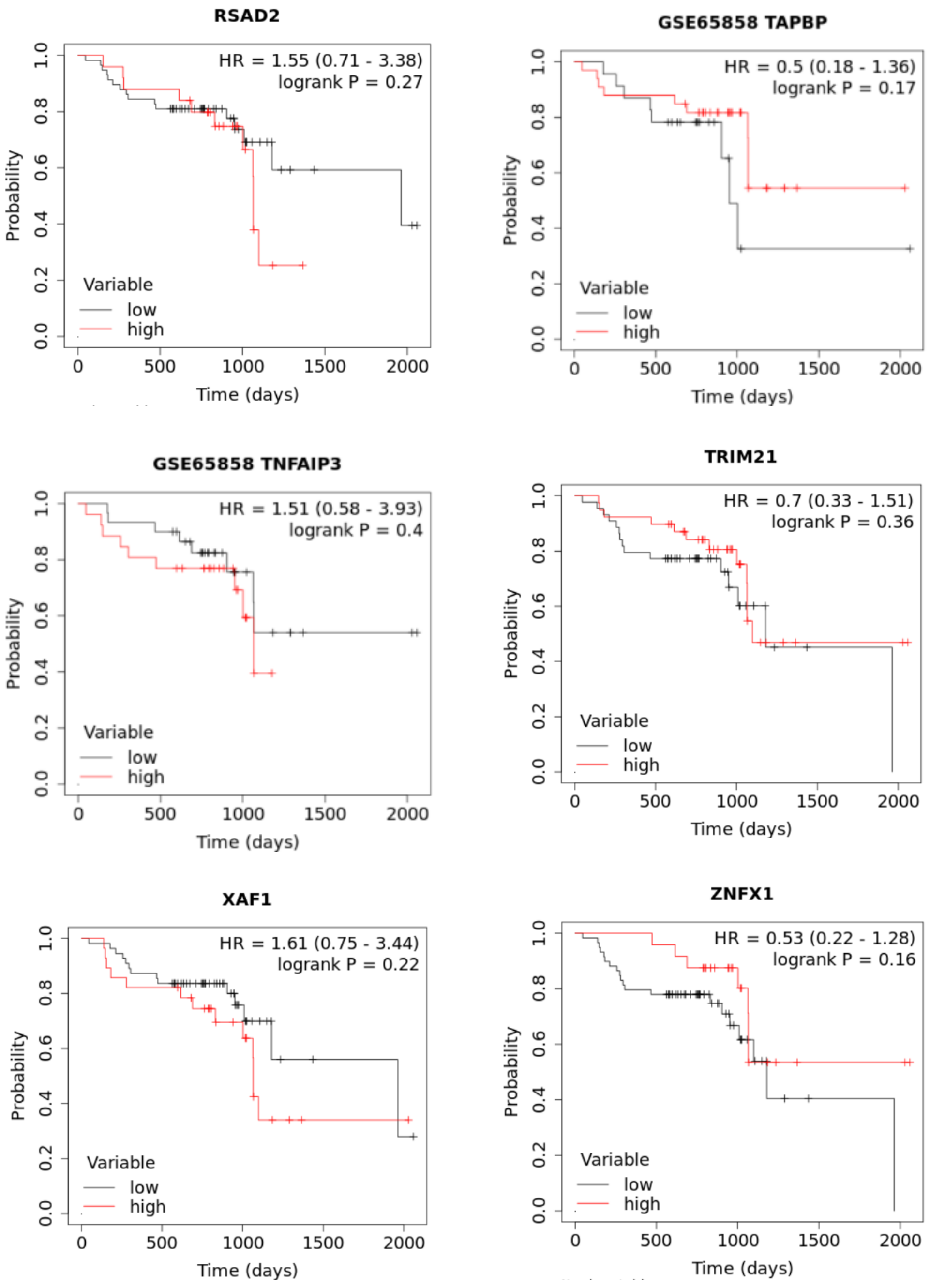

Figure S2. Kaplan-Meier plots of overall survival based on the 14 interferon pathway genes in GSE65858 data set. (Corresponding to Figure 4B).

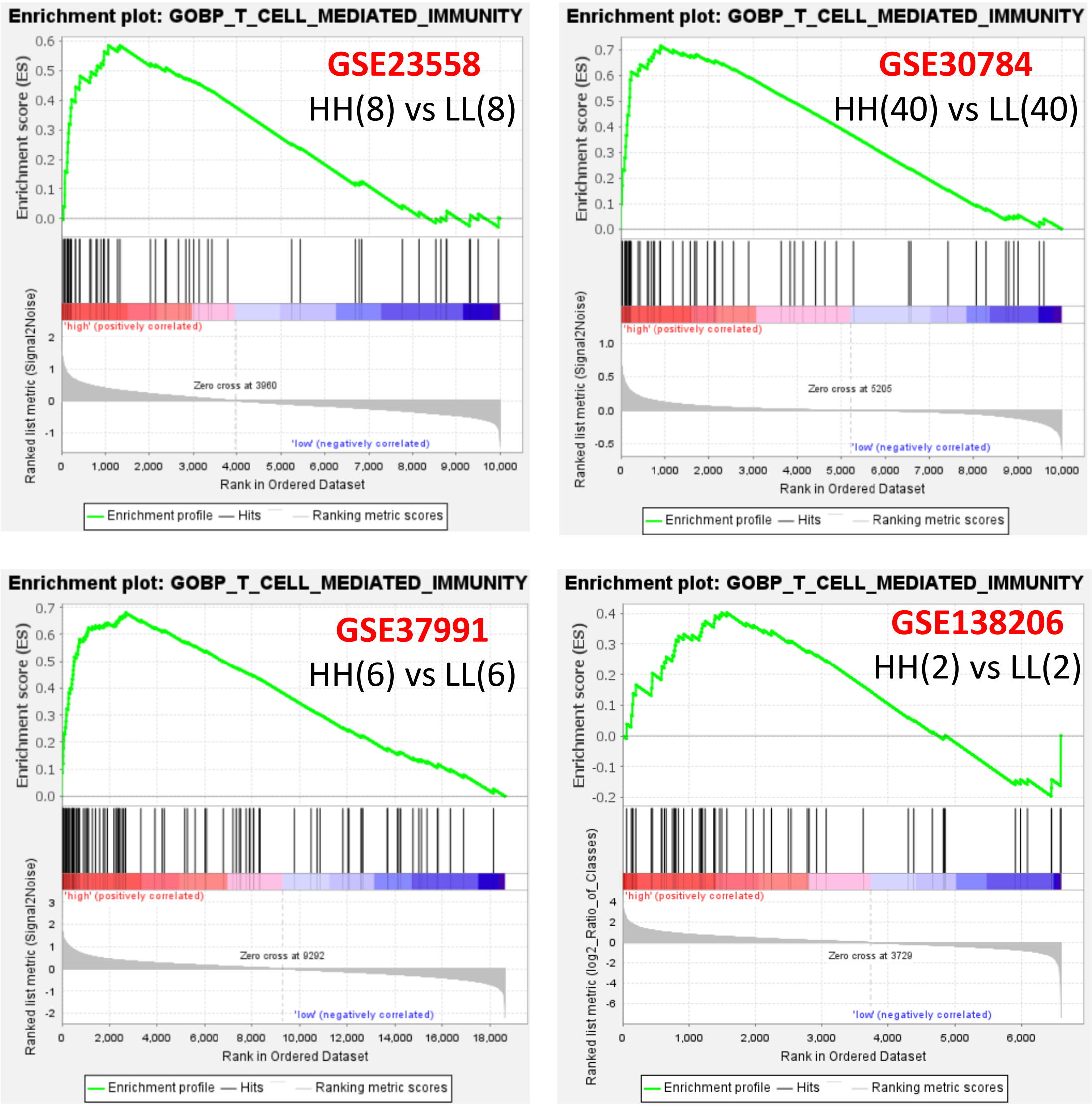

Figure S3. Enrichment plots of GOBP T-cell-mediated immunity pathway in GSE23558, GSE20784, GSE37991, and GSE138206 data sets based on the coordinated expression of CXCL10 and STAT2 (HH vs LL). The numbers in parentheses represent the numbers of patients with the most high (HH) and low (LL) expression levels of CXCL10 and STAT2, respectively, applied to gene set enrichment analysis. GOBP, Gene Ontology Biological Process. (Corresponding to Figure 6).
